# The complete chloroplast genome of *Paphiopedilum malipoense* (Orchidaceae)

**DOI:** 10.1080/23802359.2019.1642168

**Published:** 2019-07-17

**Authors:** Li-Qiang Li, Jie Huang, Li-Jun Chen, Qiang-Yong Zhang, Jian-Bing Chen

**Affiliations:** a Key Laboratory of National Forestry and Grassland Administration for Orchid Conservation and Utilization, Shenzhen, China;; b Shenzhen Key Laboratory for Orchid Conservation and Utilization, The Orchid Conservation and Research Centre of Shenzhen, The National Orchid Conservation Centre of China, Shenzhen, China

**Keywords:** *Paphiopedilum malipoense*, chloroplast genome, Orchidaceae

## Abstract

*Paphiopedilum malipoense* S.C.Chen & Z.H.Tsi is a new orchid species found in Malipo county, Yunnan. Here we report the complete chloroplast (cp) genome sequence and the features of *P. malipoense*. Its cp genome sequence of *P. malipoense* is 158,708 bp, including one large single-copy region (LSC, 89,452 bp), one small single-copy region (SSC, 17,692 bp), and two inverted repeat regions (IRs, 25,782 bp). The cp genome encoded 132 genes, of which 112 were unique genes (79 protein-coding genes, 29 tRNAs, and 4 rRNAs). The phylogenetic relationships show that *P. malipoense* is sister with *P. armeniacum-P. wardii*.

The genus *Paphiopedilum* was established by Pfitzer (1886:11), and now ∼90 species are recognized within the genus, and 27 species are distributed in China. Some new species of this genus have been published (Chen and Tsi 1984; Liu and Chen 2001, 2002, 2003; Metusala 2017). *Paphiopedilum* belongs to the subfamily Cypripedioideae (Orchidaceae), and distributes from tropical Asia to the Pacific islands, with some species extending to subtropical areas (Chen et al. 2009; Pridgeon et al. 2009). It is charactered by deeply pouched lip, staminode and lateral sepals usually fused to form a synsepal (Tsi et al. 1999). All *Paphiopedilum* species were listed in CITES I.

Leaf samples of *Paphiopedilum malipoense* were obtained from the Orchid Conservation and Research Centre of Shenzhen, and specimens were deposited in the National Orchid Conservation Center herbarium (NOCC; specimen code Z.J.Liu 7314). Complete chloroplast genome sequence of *P. malipoense* was assembled in this study. Total genomic DNA was extracted from fresh material using the modified CTAB procedure of Doyle and Doyle (1987). Sequenced on Illumina Hiseq 2500 platform (Illumina, San Diego, CA). Genome sequences were screened out and assembled with MITObim v1.8 (Hahn et al. 2013), which resulted in a complete circular sequence of 158,708 bp in length. Other sequences used in this study were downloaded from the NCBI GenBank database for phylogenetic analysis.

The cp genome sequence of *P. malipoense* (GenBank accession MN016934) is 158,708 bp length and presented a typical quadripartite structure including one large single-copy region (LSC, 89,452 bp), one small single-copy region (SSC, 17,692 bp), and two inverted repeat regions (IRs, 25,782 bp). The cp genome encoded 132 genes, of which 112 were unique genes (79 protein-coding genes, 29 tRNAs, and 4 rRNAs), The overall GC content was 36%.

To confirm the phylogenetic position of *P. malipoense*, a molecular phylogenetic tree was constructed based on the maximum-likelihood (ML) methods with eight species from *Paphiopedilum* and two species as outgroup. The ML analysis was performed using the CIPRES Science Gateway web server (RAxML-HPC2 on XSEDE 8.2.10) with 1000 bootstrap replicates and settings as described by Stamatakis et al. (2008). The results showed that *P. malipoense* is mostly related taxa with *Paphiopedilum armeniacum* and *Paphiopedilum wardii*. (Figure 1). This newly reported chloroplast genome provides a good foundation for the identification and genotyping of *Paphiopedilum* species.

**Figure 1.**
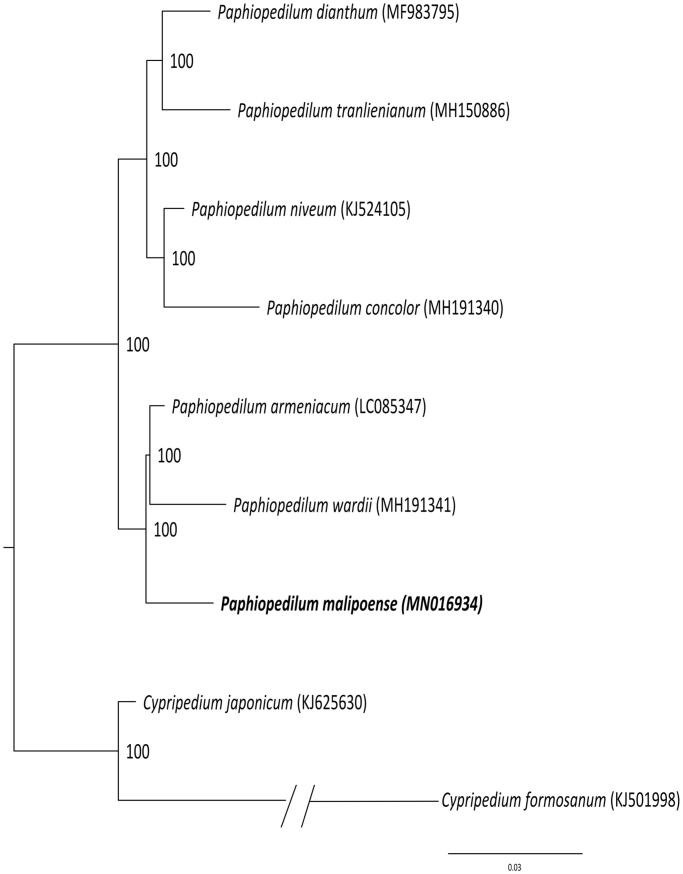
Phylogenetic position of *Paphiopedilum malipoense* inferred by maximum-likelihood (ML) of complete cp genome. The bootstrap values are shown next to the nodes.
